# Decoding the Epigenetics of Infertility: Mechanisms, Environmental Influences, and Therapeutic Strategies

**DOI:** 10.3390/epigenomes8030034

**Published:** 2024-09-05

**Authors:** Lara Saftić Martinović, Tea Mladenić, Dora Lovrić, Saša Ostojić, Sanja Dević Pavlić

**Affiliations:** 1 Department of Medical Biology and Genetics, Faculty of Medicine, University of Rijeka, 51000 Rijeka, Croatia; lara.saftic.martinovic@medri.uniri.hr (L.S.M.); tea.mladenic@medri.uniri.hr (T.M.); sasa.ostojic@uniri.hr (S.O.); 2 Faculty of Biotechnology and Drug Development, University of Rijeka, 51000 Rijeka, Croatia; dora.lovric@student.uniri.hr

**Keywords:** infertility, epigenetics, DNA methylation, histone modification, non-coding RNAs, gametogenesis, environmental factors, therapeutic interventions

## Abstract

Infertility is a complex condition caused by a combination of genetic, environmental, and lifestyle factors. Recent advances in epigenetics have highlighted the importance of epigenetic changes in fertility regulation. This review aims to provide a comprehensive overview of the epigenetic mechanisms involved in infertility, with a focus on DNA methylation, histone modification, and non-coding RNAs. We investigate the specific epigenetic events that occur during gametogenesis, with a focus on spermatogenesis and oogenesis as distinct processes. Furthermore, we investigate how environmental factors such as diet, stress, and toxin exposure can influence these epigenetic changes, potentially leading to infertility. The second part of the review explores epigenetic changes as therapeutic targets for infertility. Emerging therapies that modulate epigenetic marks present promising opportunities for fertility restoration, particularly in spermatogenesis. By summarizing current research findings, this review emphasizes the importance of understanding epigenetic contributions to infertility. Our discussion aims to lay the groundwork for future research directions and clinical applications in reproductive health.

## 1. Introduction

Infertility, defined as the inability to conceive after one year of regular, unprotected sexual intercourse, affects 8–12% of couples of reproductive age [1]. It can be caused by a variety of factors affecting both men and women, including hormonal imbalances, structural abnormalities, and genetic disorders. Infertility is usually divided into two categories: primary infertility, where pregnancy has never occurred, and secondary infertility, where pregnancy has occurred but subsequent attempts to conceive have failed [2].

Infertility can involve issues with one or both partners. Research indicates that the cause of infertility lies with the man in approximately 20–30% of cases, with the woman in 20–35% of cases, with both partners in 25–40% of cases, and remains unknown in 10–20% of cases [3,4]. The most common causes of female infertility include ovulatory disorders (25%), endometriosis (15%), pelvic adhesions (12%), tubal obstructions (11%), uterine lining abnormalities (11%), and hyperprolactinemia [5]. Ovulatory disorders, such as polycystic ovary syndrome (PCOS), primary ovarian insufficiency (POI), and hypothalamic dysfunction, can prevent the release of a viable egg. Alternatively, tubal obstructions, often caused by pelvic inflammatory disease, endometriosis or previous surgeries, can prevent the egg from passing through the fallopian tubes and meeting the sperm, while fibroids, polyps, and congenital malformations in the uterus can prevent a fertilized egg from implanting properly [5]. An age-related decline in egg quality, hormonal imbalances, and autoimmune diseases also contribute significantly to infertility. Moreover, environmental toxins, high stress levels, obesity, and lifestyle choices such as smoking and alcohol consumption can negatively impact female fertility [5]. Additionally, certain medical conditions, such as thyroid disorders, diabetes, autoimmune diseases, and weight issues (being both underweight and overweight), further influence a woman’s fertility.

On the other hand, male infertility is most commonly caused by issues with sperm production, function, or release, resulting in low sperm count (oligozoospermia), poor sperm motility (asthenozoospermia), abnormal sperm morphology (teratozoospermia), or a complete absence of sperm in ejaculate (azoospermia). Factors contributing to male infertility include testicular damage or diseases, hormonal imbalances, genetic conditions, and blockages in the reproductive tract. Additionally, environmental factors such as exposure to toxins, excessive heat, obesity, and substance abuse can negatively impact male fertility. With advances in medical science, understanding the underlying causes of infertility and developing effective treatments remains a priority [6,7,8].

Epigenetics refers to heritable changes in gene function that do not involve alterations in the nucleotide sequence of the DNA molecule. These changes impact fertility by regulating genes that are important for reproduction. Environmental factors, lifestyle choices, and even parental age can induce epigenetic changes that affect reproductive health, potentially reducing the chances of successful conception and healthy embryo development. Understanding these epigenetic influences can lead to more accurate diagnoses, personalized treatments, and improved fertility outcomes [3,6,8].

This review aims to provide a comprehensive analysis of the complex relationship between epigenetic mechanisms and infertility. The first part examines basic epigenetic modifications, such as DNA methylation, histone modifications, and non-coding RNAs, explaining their effects on fertility. The following section organizes the various influences of environmental factors, including endocrine-disrupting chemicals, diet, lifestyle choices, and pollutants, detailing how they affect fertility by altering epigenetic modifications. Specific epigenetic modifications in male and female infertility are then discussed, focusing on research findings and comparative analyses. The review also covers current diagnostic methods for detecting these changes and potential therapeutic strategies targeting epigenetic mechanisms, with an emphasis on recent advances in epigenetic therapies. Finally, it highlights the crucial role of epigenetics in understanding the complex interplay between genes and environment in reproductive health, and how this knowledge can improve diagnostics and personalized treatments, ultimately enhancing our ability to more effectively address and treat infertility.

## 2. Epigenetic Mechanisms in Infertility

Epigenetic mechanisms form a complex network of biochemical modifications that surround DNA molecules and modulate genomic function without altering the nucleotide sequence. By regulating gene expression, these modifications control phenotypic outcomes without necessitating genetic alterations. DNA methylation, post-translational histone modifications, and non-coding RNA regulatory activity are all key epigenetic processes that contribute to the dynamic architecture of chromatin and gene accessibility. Epigenetic reprogramming during gametogenesis ensures the proper development and maturation of gametes by resetting and establishing specific epigenetic marks. The establishment of distinct epigenetic landscapes during embryogenesis is critical for directing cell differentiation and tissue formation, thereby facilitating successful embryo implantation and development. Deviations in these epigenetic processes can lead to reproductive disorders, highlighting the importance of accurate epigenetic regulation in preserving reproductive health [3].

### 2.1. DNA Methylation

The most studied and commonly observed epigenetic mechanism is DNA methylation, which occurs on the cytosine of CpG dinucleotides following replication [9]. This process involves the addition of a methyl group from the donor S-adenosylmethionine (SAM) to the fifth carbon atom (C5) of cytosine residues within CpG dinucleotides, creating 5-methylcytosine (5mC). A CpG dinucleotide consists of cytosine linked by a phosphodiester bond to guanine and is most often found in clusters known as CpG islands (CGIs). These CGIs are typically associated with the promoters and regulatory regions of genes and play a key role in transcription initiation. DNA methylation frequently silences gene expression, whereas unmethylated genes are active or can be activated.

Enzymes that catalyze the transfer of a methyl group to DNA are known as DNA methyltransferases (DNMTs). DNMT1 is primarily responsible for maintaining existing methylation patterns by copying them from parental DNA strands to newly synthesized strands, thereby ensuring the transfer of epigenetic information during cell division [10]. On the contrary, DNMT3a and DNMT3b are specialized in establishing new methylation patterns (de novo methylation) at previously unmethylated CpG sites, which is particularly important during early development and cell differentiation because it influences cellular identity via epigenetic changes [10]. Moreover, DNMT2, DNMT3c, and DNMT3l are also involved in different methylation processes: DNMT2 participates in RNA methylation pathways, particularly in embryonic stem cells, demonstrating the diverse roles of DNMT family members in cellular regulation; DNMT3c methylates retrotransposon promoters in male germ cells, thereby silencing these potentially disruptive elements and protecting genome stability; DNMT3l functions as a coactivator essential for establishing maternal genomic imprints during oocyte development and participates in spermatogenesis [9,11].

Apart from the enzymes that establish the methylation patterns, DNA methylation also involves the opposite process—DNA demethylation. During DNA demethylation, 5mC is converted back to cytosine through either active or passive mechanisms. The active mechanism involves the ten-eleven translocation (TET) family of enzymes, which catalyzes the hydroxylation of 5mC, followed by activation-induced cytosine deamination. In contrast, passive demethylation occurs due to insufficient availability of the SAMs, a global methyl donor, which inhibits the action of the DNMT1 enzyme.

When DNA is methylated, proteins with methyl binding domains (MBDs) bind to it, including MeCP2 (methyl CpG binding protein 2), MBD1, MBD2, and MBD4. This further inhibits transcription factor recruitment to DNA, resulting in reduced gene expression. DNMT1, DNMT3a, DNMT3b, DNMT3c, and DNMT3l are essential components of this complex regulatory system [9,11]. It is important to note that while a gene might exhibit overall hypomethylation, certain regulatory regions within the gene can be hypermethylated, leading to complex patterns of gene expression dysregulation.

#### 2.1.1. DNA Methylation in Male Infertility

DNA methylation ensures proper gene expression necessary for spermatogenesis and plays a critical role in male fertility; thus, any abnormalities in DNA methylation patterns can impair sperm development and function, ultimately contributing to infertility. Previous research has identified altered DNA methylation patterns in numerous genes that are critically associated with infertility. These findings suggest that aberrations in DNA methylation can disrupt the expression of key genes involved in reproductive processes, thereby contributing to the pathogenesis of infertility (Table 1) [11,12,13,14,15,16,17,18,19]. For example, changes in DNA methylation have been observed in the promoter region of the methylenetetrahydrofolate reductase (MTHFR) gene alongside hypomethylation in the IGF2-H19 locus, and hypermethylation in genes such as MEST and SNRPN [12,13].

The abnormal methylation of the MTHFR gene, which plays a critical role in DNA and folate synthesis, as well as in the DNA methylation process itself, has been extensively studied [14]. Inactivating MTHFR in mice results in the hypomethylation of sperm DNA and an arrest of spermatogenesis, highlighting its crucial role in reproductive processes [15]. MTHFR gene mutations decrease enzyme activity, resulting in reduced methionine availability and impaired DNA methylation. Additionally, hypermethylation of the MTHFR gene can reduce its activity, negatively impacting the methylation status of sperm DNA.

Moreover, Tang et al. investigated epigenetic dysregulation in the H19, GNAS, and DIRAS3 imprinted genes, which are important for growth regulation and development via parent-of-origin expression [16]. The paternally imprinted and methylated differentially methylated region (DMR)s of H19 showed hypomethylation, whereas the maternally imprinted and unmethylated DMRs of GNAS and DIRAS3 showed increased methylation in idiopathic infertile males. This suggests that hypo- and hypermethylation occur simultaneously in the haploid sperm genome. Abnormal methylation at the H19 DMR was found in 19.3% of patients, including one case with no methylation. However, patients with completely unmethylated H19 did not have abnormal methylation of GNAS or DIRAS3. These findings suggest that epigenetic alterations in these imprinted genes may serve as potential biomarkers for diagnosing male infertility [16]. However, idiopathic infertile males, particularly those with oligozoospermia, tend to have lower global methylation levels. This means that, while certain regions or genes may exhibit hypermethylation, the overall methylation level across the genome may be lower. This lower global methylation may be indicative of a broader epigenetic dysregulation in idiopathic male infertility. Indeed, Li et al. revealed that control individuals exhibited 100% methylation of H19 imprinting control region (ICR), while infertile males with asthenozoospermia and oligozoospermia showed hypomethylation (a methylation status of around 94%) of H19, with the average methylation rates of CpG 1, 3, and 6 in the infertile group being statistically different from those in the normal control group [17]. Another study identified 2752 CpGs with abnormal DNA methylation patterns, which were significantly associated with CpG sites that were specifically methylated in sperm versus somatic cells [18]. DNA hypomethylation was linked to regions with high levels of H3K4me1 and CCCTC-binding factor (CTCF), a zinc finger protein that regulates chromatin’s 3D structure. CTCF is involved in chromatin loop formation, insulator function, and the maintenance of topologically associating domain (TAD) boundaries, implying a locus-dependent link between chromatin context and abnormal DNA methylation. Furthermore, sperm samples from infertile patients demonstrated significantly lower DNA methylation levels at several repetitive sequences, including LINE-1, Alu Yb8, NBL2, and D4Z4, compared to somatic cells and controls [18]. Boissonass et al. investigated the DNA methylation status in human sperm, focusing on the IGF2-H19 locus. IGF2 (Insulin-like Growth Factor 2) and H19 are two genes that are closely linked (on chromosome 11) and subject to genomic imprinting. The study analyzed 47 CpGs located in DMRs of the IGF2 gene (DMR0 and DMR2) and the H19 gene (third and sixth CTCF-binding sites) using pyrosequencing. Results showed that in the teratozoospermia group, around 60% of the patients had a loss of methylation at variable CpG positions, particularly in the IGF2 DMR2 and/or the sixth CTCF-binding site of the H19 DMR. Similarly, in the oligoasthenoteratozoospermia group, around 73% of patients exhibited severe methylation loss at the sixth CTCF site, which was closely related to sperm count. These findings suggest that the sixth CTCF site of the H19 DMR is a significant biomarker for quantitative defects in spermatogenesis [20]. A study by Navarro-Costa et al. revealed that oligoasthenoteratozoospermic patients exhibit significantly higher methylation defects in the DAZL promoter CGI compared to normozoospermic controls, with unmethylated DAZL clone frequencies dropping from 100% in normozoospermic men to 81% and 62% in normal and defective germ cell-enriched fractions of oligoasthenoteratozoospermic patients, respectively. In contrast, the DAZ promoter CGI remained consistently unmethylated across all groups, maintaining over 97% unmethylation. Significant differences in DNA methylation profiles were also observed within both normozoospermia and oligoasthenoteratozoospermia groups when comparing normal sperm-enriched fractions to defective germ cell-enriched fractions, indicating more pronounced epigenetic disturbances in the latter. The highest epigenetic distances were recorded between normal sperm-enriched fractions from normozoospermic men and any fraction from oligoasthenoteratozoospermic men, underscoring substantial epigenetic heterogeneity [19].

#### 2.1.2. DNA Methylation in Female Infertility

Impaired DNA methylation contributes significantly to female infertility by disrupting the normal regulation of gene expression required for reproductive health. Abnormal methylation patterns, such as hypermethylation or hypomethylation, can cause dysregulation of genes involved in important reproductive processes. Table 2 summarizes how impaired DNA methylation affects fertility in females, highlighting specific genes or regions, the nature of methylation changes, and their impact on reproductive outcomes.

Shacfe et al. in their review paper summed up all methylation changes that occur in female infertility [15]. Hypermethylation in the HOXA10 promoter region, which is required for endometrial receptivity and implantation, reduces expression and contributes to endometriosis and infertility. In PCOS, hypomethylation of the CYP19A1 gene promoter increases estrogen biosynthesis, resulting in hormonal imbalances that impair ovarian function. Similarly, hypomethylation in the promoter regions of the FKBP5 and YAP1 genes causes their overexpression, which disrupts ovarian health and cell function, respectively. The LHCGR gene, which is involved in hormone signaling, is also hypomethylated in PCOS, affecting hormonal signaling and fertility. Endometriosis also causes hypermethylation in the promoter regions of the PR (progesterone receptor) and ESR1 (estrogen receptor 1) genes, which are essential for steroid hormone signaling and endometrial function, reducing their expression and impairing endometrial receptivity. Additionally, KDM6A, a histone demethylase involved in chromatin modification, exhibits hypomethylation in its promoter region, affecting chromatin structure and gene expression, which is associated with Turner syndrome. USP9X, a deubiquitinating enzyme, also has hypomethylation in its promoter region, which influences protein degradation pathways in Turner syndrome. The ZFX gene, which is involved in cell differentiation and proliferation, exhibits hypomethylation in its promoter region, affecting transcription regulation in Turner syndrome [15].

Uysal et al. found a significant decrease in global DNA methylation levels in postnatal mouse ovaries as they age. This reduction is associated with decreased expression of DNMT1, DNMT3a, and DNMT3l, suggesting that these changes may contribute to the decline in female fertility during ovarian aging [21]. DNMTs were found in a variety of ovarian cell types, including oocytes, granulosa cells, theca cells, luteal cells, and stromal cells, with significant decreases in older ovaries. The intensity of 5-methylcytosine (5mC) staining, which measures global DNA methylation levels, was highest in young ovaries and lowest in older ovaries, supporting the hypothesis that decreased DNA methylation is a marker of ovarian aging and reduced fertility. Reduced DNMT expression and global DNA methylation are likely to disrupt the regulation of genes required for follicular growth and oocyte maturation, leading to various human reproductive disorders [21,22].

Tang et al. investigates the link between sleep quality and infertility in women of childbearing age, with a focus on the mediating role of DNA methylation [23]. The study found 262 differentially methylated CpG sites corresponding to 185 genes in women with anovulatory infertility compared to healthy controls. Of these, 180 CpG sites were hypermethylated and 82 were hypomethylated. A specific CpG site, cg08298632, on the KCNC2 gene, was discovered to play a positive mediating role in the link between difficulty falling asleep and infertility. The mediating effect coefficient for this site was 0.10 (95% CI = 0.01–0.22), and this methylation site mediated 64.3% of the total effect [23].

### 2.2. Histone Modifications

Histones are essential proteins that organize DNA into nucleosomes, the fundamental units of chromatin. The charged N-terminal region of the histone, known as the histone tail, comprises 15 to 38 amino acids whose modifications influence the compaction of nucleosomes into more condensed chromatin structures. Each nucleosome consists of 145–147 base pairs of DNAs wrapped around a core histone protein composed of an octamer formed by pairs of H2A, H2B, H3, and H4 histones. This octamer is linked by histone H1, which completes the nucleosome facilitating its condensation into higher-order structures. In its condensed state, chromatin remains folded, resulting in stacked nucleosomes that are not easily accessible for gene activation [24]. Considering the degree of condensation, chromatin can be divided into euchromatin and heterochromatin. Euchromatin is characterized by loosely packed nucleosomes with wider spacing between them, allowing for active transcription, while heterochromatin is highly condensed and therefore transcriptionally inactive.

Covalent modifications to histone tails, such as acetylation, methylation, phosphorylation, and ubiquitination, can affect histone–DNA interactions. The most common histone modifications are methylation and acetylation. Acetylation involves adding acetyl groups to lysine residues, which decreases the positive charge on histones and weakens histone–DNA interactions while increasing gene accessibility. In contrast, methylation involves the addition of methyl groups to lysine or arginine residues. Since lysine and arginine are the most abundant amino acids in histones, they are frequently subject to acetylation and/or methylation. Acetylation predominantly occurs in uncondensed chromatin, facilitating gene activation. Methylation, however, can be present in both condensed and uncondensed chromatin, with its effect depending on the specific site and degree of methylation (mono-, di- or tri-methylation) [24].

Enzymes involved in histone modification are classified into three types: writers, erasers, and readers. Writers are enzymes that add specific modifications, such as histone acetyltransferases (HATs) for acetylation and histone methyltransferases (HMTs) for methylation. Erasers are enzymes that remove these modifications, including histone deacetylases (HDACs) for deacetylation and histone demethylases (HDMs) for demethylation. Readers are proteins that recognize and bind to specific histone modifications, thereby altering the structure and function of chromatin [24].

#### 2.2.1. Histone Modifications in Male Infertility

During spermatogenesis, histone H4 undergoes hyperacetylation at specific lysine residues. These acetylation events are required for nucleosome destabilization and remodeling, which facilitate the replacement of histones with protamines in the elongating spermatids nucleus during their maturation. This transition is necessary for chromatin condensation and sperm head formation. Studies have shown that decreased levels of histone H4 acetylation are significantly associated with impaired spermatogenesis, leading to conditions such as oligozoospermia and azoospermia [25].

Abnormal histone methylation can impair the process of spermatogenesis, emphasizing the critical role of enzymes involved in maintaining histone methylation patterns, specifically HDMs, which are particularly important during meiosis, as demethylation is necessary for the continuation of spermatogenesis [26]. Specific methylation marks, such as H3K4me and H3K27me, are essential for controlling gene expression during this process [27]. Men who are infertile have been found to exhibit hypomethylation at these residues, indicating that proper control of histone methylation is necessary for healthy sperm development. Moreover, chromatin condensation defects and consequent male infertility can result from the loss of JHDM2A, a histone demethylase specific for H3K9me2/1 [28]. This indicates that precise regulation of histone methylation is essential for successful spermatogenesis and the production of viable sperm [25].

#### 2.2.2. Histone Modifications in Female Infertility

Equally significant to female fertility are histone modifications, especially during the growth and maturation of oocytes. Histone acetylation and methylation regulate the expression of several genes essential for oogenesis. For instance, chromatin remodeling during oocyte maturation depends on H4 acetylation and H3 acetylation at lysine 9 [29]. These changes ensure that the chromatin is sufficiently loosened to permit the transcription of genes required for oocyte development. Aberrant acetylation patterns, such as hypoacetylation of H3K9 and H4, can result in chromatin condensation defects, which impair the oocytes’ ability to develop and ultimately lead to infertility.

Additionally, site-specific methylation of histones, such as H3K27me3 and H3K4me3, is important for regulating gene expression during oogenesis [30]. These modifications help maintain the delicate balance of gene activation and repression required for proper oocyte development. Dysregulation of histone methylation can impair oocyte quality and cause developmental arrest.

### 2.3. Non-Coding RNAs

Non-coding RNAs (ncRNAs) include functional RNA molecules that do not encode proteins but exert various regulatory functions within the cell. Beyond their roles in regulating transcriptional and post-transcriptional processes, they play an important role in the epigenetic regulation of gene expression. RNAs with regulatory functions are classified into two types based on size: small ncRNAs (including miRNAs, siRNAs and piRNAs) and long ncRNAs (lncRNAs) [31].

#### 2.3.1. Small ncRNAs

MicroRNAs (miRNAs) are single-stranded, non-coding RNA molecules that range from 19 to 25 nucleotides in length. To date, over 2000 different miRNAs have been identified in the human genome, and they are estimated to regulate the activity of up to one-third of all human genes, highlighting their profound significance in gene regulation [32]. Their mechanism of action involves interacting with numerous transcriptional and epigenetic regulators within cells, thereby modulating the expression levels of multiple genes. Together with small interfering RNAs (siRNAs), short double-stranded RNAs (20–25 nucleotides), miRNAs are involved in post-transcriptional gene regulation through the mechanism of RNA interference. This process involves complementary binding to target mRNA molecules, resulting in their degradation via the RNA-induced silencing complex (RISC) or inhibition of the translation process [33]. In addition to miRNAs and siRNAs, another class of short ncRNAs involved in epigenetic regulation is Piwi-interacting RNAs (piRNAs). piRNAs are short RNA molecules (26–31 nucleotides in length) that play important roles in transposon methylation and chromatin regulation (transposon silencing). They interact with Piwi proteins, playing a crucial role in silencing specific genes. For example, they are known to target homeobox genes that regulate developmental processes during embryonic development [34]. Sperm maturation and function are dependent on miRNAs, which regulate genes involved in cell cycle progression and apoptosis, ensuring that spermatocytes develop properly and produce mature sperm. Wang et al. presented markedly altered miRNAs in infertile patient groups compared to controls. In detail, the authors confirmed that 7 miRNAs (miR-34c-5p, miR-122, miR-146b-5p, miR-181a, miR-374b, miR-509-5p, and miR-513a-5p) were significantly decreased in azoospermia but increased in asthenozoospermia [35].

Understanding how microRNAs (miRNAs) affect the release of ovarian steroid hormones (progesterone, testosterone, and estradiol) is important in infertility research for a variety of reasons [36]. miRNAs such as miR-24, miR-25, miR-132, miR-182, miR-18, miR-15, and miR-32 specifically suppress estradiol release, suggesting that they may play an inhibitory role in ovarian function. In contrast, miRNAs such as miR-107, miR-150, miR-151, and miR-152 do not increase estradiol release, indicating that they may be involved in ovarian follicle viability rather than hormone secretion. Furthermore, miRNAs such as miR-125 have been shown to influence cholesterol metabolism, which affects steroidogenesis. Therefore, understanding the specific roles of these miRNAs is crucial for the development of future therapeutic strategies to modulate fertility depending on the desired outcome [36].

Moreover, piRNAs have a crucial role in various stages of spermatogenesis, including the establishment of DNA methylation patterns, and sperm production [37]. The human Piwi protein subfamily includes PIWIL1 (HIWI), PIWIL2 (HILI), PIWIL3 (HIWI3), and PIWIL4 (HIWI2). Gu et al. have hypothesized that genetic variations in genes encoding these proteins might impact normal spermatogenesis. In a case-control study of male infertility in a Han Chinese population, they genotyped these polymorphisms and found that an SNP in the 3′untranslated region of HIWI2 and a non-synonymous SNP in HIWI3 increase the risk of oligozoospermia. Conversely, a non-synonymous SNP in PIWIL3 was associated with reduced risk, while a common haplotype of PIWIL4 significantly lowered the risk of oligozoospermia [38].

tRNA-derived small RNAs (tsRNAs) are fragments that originate from precursor or mature transfer RNAs. They have received attention for their ability to regulate gene expression and contribute to epigenetic modifications. Zhu et al. have confirmed that tsRNAs regulate the expression of genes involved in sperm maturation and motility, which affect male fertility [39]. Specifically, tsRNAs can influence DNA methylation patterns and histone modifications, both of which are required for normal sperm cell growth. During early embryogenesis in females, tsRNAs govern the expression of retrotransposons and other genetic components. Improper tsRNA regulation can result in aberrant epigenetic marks, potentially leading to developmental defects in offspring [40].

Ribosomal RNA-derived short RNAs (rsRNAs) are derived from ribosomal RNA and have been linked to gene control, namely through interactions with Argonaute proteins. These short RNAs can influence a cell’s translational machinery, affecting the entire epigenetic landscape. According to Derakhshan et al., rsRNAs can influence gene expression during gametogenesis by modifying chromatin shape and accessibility [41]. This modulation is critical throughout gamete development because it ensures correct gene expression, which is essential for successful fertilization and early embryo development.

Small nuclear RNAs (snRNAs) and small nucleolar RNAs (snoRNAs) are best known for their involvement in RNA processing, which include splicing and rRNA modification, respectively. However, their influence extends to the regulation of epigenetic changes as well. In male fertility, snRNAs and snoRNAs have been linked to genomic stability during spermatogenesis. Specifically, snoRNAs guide chemical alterations that stabilize chromatin structure and affect gene expression during sperm formation [41]. In females, snRNAs and snoRNAs help oocytes develop properly by ensuring precise splicing and rRNA modification, both of which are required for chromatin remodeling and gene expression during oocyte maturation.

Pseudogene-derived small interfering RNAs (endo-siRNAs) are a type of small RNA produced by pseudogenes and transposons. Endo-siRNAs serve an important function in genomic integrity by silencing transposable elements and regulating gene expression. Endo-siRNAs have a crucial role in male fertility by maintaining the epigenetic integrity of sperm cells. They inhibit the activation of transposons, which can cause genomic instability and reduced fertility [39]. In females, endo-siRNAs regulate oocyte transcripts, affecting the epigenetic landscape that is crucial for early embryonic development. Incorrect expression of endo-siRNAs can cause epigenetic reprogramming, leading to reproductive difficulties and developmental abnormalities in children [41].

#### 2.3.2. Long ncRNAs

Long ncRNAs (lncRNAs) are ncRNA molecules that are more than 200 nucleotides long and reside in the nucleus or cytoplasm. Key examples of lncRNAs involved in gene silencing include H19 and Xist RNAs. Their interactions with small ncRNAs, such as siRNAs, illustrate the complex network of chromatin regulation and gene silencing mechanisms involving various non-coding RNAs.

XIST is a lncRNA required for X-chromosome inactivation in females [42]. It inactivates the X chromosome and recruits’ chromatin-modifying complexes, leading to heterochromatin formation and gene silencing. The proper function of XIST ensures the appropriate dosage of X-linked gene expression. Abnormal XIST expression or function can disrupt X-chromosome inactivation, potentially affecting ovarian function and fertility.

H19 is an imprinted lncRNA expressed from the maternal allele that plays a crucial role in regulating genomic imprinting and chromatin structure [43]. It interacts with chromatin-modifying proteins and miRNAs to influence gene expression during embryonic development and is essential for proper development of the placenta and embryo. A study by Peng et al. highlighted the diverse roles of H19 in various conditions, including PCOS, endometriosis, uterine fibroids, diminished ovarian reserve (DOR), male infertility, and issues related to assisted reproductive technologies (ART) [44]. In PCOS, H19 is implicated in regulating reproductive and metabolic abnormalities, affecting hyperandrogenemia and granulosa cells proliferation. In endometriosis, H19 levels fluctuate with the menstrual cycle and influence cell proliferation and invasion via pathways involving miR-124-3p and integrin beta 3. In uterine fibroids, H19 contributes to extracellular matrix deposition and cell proliferation through pathways involving Let7 and TET3. In cases of DOR, reduced levels of H19 correlate with decreased ovarian reserves and altered steroid hormone production. In male infertility, hypomethylation of H19 is associated with conditions such as oligozoospermia and asthenozoospermia. Finally, in ART-related pathologies, H19 affects endometrial receptivity and implantation success. Metastasis Associated Lung Adenocarcinoma Transcript 1 (MALAT 1) is an lncRNA involved in gene expression regulation, RNA processing, and chromatin dynamics [45]. It is crucial for fertility, particularly in spermatogenesis and overall reproductive health, by maintaining genomic stability in sperm cells through oxidative stress response regulation and DNA repair mechanisms. Reduced MALAT1 expression in infertile men is associated with increased DNA damage in sperm, highlighting its importance in maintaining sperm quality [46].

Similarly, decreased HOX Transcript Antisense RNA (HOTAIR) expression in infertile men correlates with increased DNA fragmentation and oxidative stress markers [46].

Steroid Receptor RNA Activator (SRA) functions as both an RNA molecule regulating transcriptional factors and a coactivator of nuclear receptors like androgen and estrogen receptors [47]. Abnormal SRA expression affects the PI3K/Akt and MAPK pathways crucial for ovarian follicle development and spermatogenesis. Hyper-methylation of the SRA gene is associated with impaired sperm parameters, contributing to conditions like non-obstructive azoospermia [47].

Maternally Expressed 3 (MEG3), imprinted gene, located on chromosome 14q32, has similar effects as H19. Atypical imprinted information could affect the motility, morphology, and concentration of sperm [48]. MEG3 influences gene expression by modulating DNA methylation and histone modifications. It enhances p53 activity, vital for cell cycle regulation and apoptosis in ovarian function. MEG3 also regulates metabolic pathways essential for energy homeostasis and steroidogenesis, and its altered expression due to in utero environmental factors can program future reproductive health. Hyper- or hypomethylation of MEG3 is linked to reproductive disorders like PCOS and POI, highlighting its role in infertility [49].

## 3. Environmental Influences on Epigenetics and Infertility

Exposure to numerous environmental pollutants, including endocrine-disrupting chemicals (EDCs), different types of diet and nutrition, as well as various lifestyle factors (e.g., stress, smoking, and alcohol consumption), collectively contribute to infertility through their effects on epigenetic mechanisms.

### 3.1. Environmental Pollutants

The impact of various environmental factors on epigenetic changes has significant implications for infertility. This section explores how different influences, including environmental pollutants and lifestyle factors, affect epigenetic mechanisms and subsequently fertility.

#### 3.1.1. Endocrine-Disrupting Chemicals

EDCs are a diverse group of chemical compounds, both synthetic and natural, that disrupt the endocrine system. Vessa et al. have reviewed the effects of common EDCs, such as pesticides and plasticizers, on female fertility, focusing on compounds like dichlorodiphenyldichloroethylene (DDE), hexachlorobenzene (HCB), hexachlorocyclohexane (HCH), dichlorodiphenyltrichloroethane (DDT), bisphenol A (BPA), di(2-ethylhexyl) phthalate (DEHP), monoethylphthalate (MEP), monobutylphthalate (MBP), and mono(3-carboxypropyl) phthalate (mcPP) [50]. DDE exposure from contaminated food, water, air, dust, and soil was linked to lower ART clinical pregnancy and live birth rates, decreased fecundity, reduced fertilization rates, fewer high-quality embryos, and an increased risk of miscarriage. Similarly, exposure to β-HCH and HCB was linked to lower fecundity, decreased fertilization rates, and fewer high-quality embryos, with β-HCH also increasing the likelihood of miscarriage. DDT exposure was specifically associated with an elevated risk of miscarriage, while BPA, present in contaminated food, consumer products, and packaging, reduced oocyte yield, inhibited normal fertilization, and increased the risk of miscarriage. DEHP, found in medical devices, cleaning supplies, and cosmetics, was linked to lower oocyte yields, clinical pregnancy rates, and live birth rates. Finally, exposure to MEP and MBP was associated with reduced likelihood of normal fertilization and overall fertility [50]. When talking about specific epigenetic mechanisms, those which cause dysregulation are responsible for severe outcomes. For instance, BPA exposure increases miR-29 expression, which subsequently decreases DNA methyltransferases (DNMT1, 3a, and 3b) mRNA levels, causing hypomethylation [51]. BPA also decreases HDAC enzyme expression. Another EDC, diethylstilbestrol (DES) reduces the lysine acetyltransferase 2A (KAT2A) expression, affecting genes critical for hormone regulation and reproductive function. HOTAIR, a long non-coding RNA, is extensively studied for its fertility-related effects. Exposure to BPA and DES increases HOTAIR expression, altering DNA methylation patterns. Additionally, EDCs like BPA and DES can alter the expression of miRNAs such as miR-206, miR-155, miR-125b, miR-144, and miR-21 [51].

#### 3.1.2. Heavy Metals

Heavy metals like arsenic, cadmium, lead, and mercury negatively impact fertility by disrupting endocrine function and causing oxidative stress. Table 3 summarizes the negative impacts of heavy metals on fertility and their associated epigenetic changes [52,53,54,55,56,57].

Arsenic affects male reproductive health by triggering DNA methylation, deregulating spermatogenesis, and decreasing sperm quality [57], notably increasing H3K9 trimethylation, which suppresses key steroidogenic genes and reduces hormone production [56]. Cadmium lowers testosterone levels and sperm quality by impacting Sertoli and Leydig cells [53,55], increasing global DNA methylation, and causing genomic instability. [56]. Lead exposure reduces reproductive hormones like LH and FSH, leading to irregular menstrual cycles and impaired spermatogenesis [52]. Mercury accumulates in reproductive organs, causing direct damage to oocytes and sperm cells by producing ROS, resulting in increased oxidative stress and DNA damage [53]. Prenatal exposure to methylmercury is linked to DNA methylation changes at specific CpG sites, affecting gene expression and reproductive health [54].

### 3.2. Diet and Nutrition

Diet and nutrition have a direct impact on fertility because specific nutrients influence reproductive health in both men and women.

Folate, a B-vitamin, is essential for the one-carbon metabolism pathway, which regulates gene expression and genome stability through DNA methylation. In detail, adequate folate intake ensures proper methylation patterns, reducing the risk of sperm DNA fragmentation in males, which improves sperm quality. In females, folate enhances oocyte quality by maintaining proper DNA methylation during egg maturation, which is crucial for embryo development.

Omega-3 fatty acids affect the expression of genes involved in inflammation and cell membrane integrity via histone modification and other epigenetic mechanisms. In males, this improves sperm motility and morphology by keeping the sperm cell membranes fluid. Omega-3s improve ovarian function and oocyte quality in women by lowering ovarian inflammation and oxidative stress.

Antioxidants protect against oxidative stress, which can damage DNA and alter epigenetic marks. Zinc, found in meat, shellfish, and seeds, is essential for spermatogenesis, testosterone production, and regulating ovulation and menstrual cycles by supporting DNA methyltransferases. Selenium, present in Brazil nuts and seafood, is crucial for sperm motility, reduces miscarriage risk, and preserves sperm DNA integrity through selenoproteins, which protect against oxidative damage. Vitamin D, obtained from sunlight, fatty fish, and fortified foods, is vital for hormone regulation, improving sperm quality and ovarian function. Selenium also modulates DNA methylation and histone modifications, affecting chromatin structure and gene expression by inhibiting DNMTs and HDAC activity [58].

In contrast, diets high in trans fats, sugar, and processed foods can cause insulin resistance and inflammation, both of which have a negative impact on hormone balance and fertility. Obesity is linked to altered DNA methylation patterns. In obese people, genes involved in metabolism, inflammation, and oxidative stress, such as leptin (LEP), insulin-like growth factor 2 receptor (IGF2R), and hypoxia-inducible factor 3a (HIF3A), have abnormal DNA methylation. For example, LEP, a key gene in energy homeostasis, is frequently hypermethylated in obese individuals, resulting in reduced expression and disrupted metabolic regulation [59]. In addition, miRNAs involved in metabolic regulation, such as miR-103 and miR-107, are often upregulated in obesity, leading to altered insulin signaling and lipid metabolism [59]. These changes contribute to increased oxidative stress, which exacerbates hormonal imbalances. Elevated oxidative stress levels can damage reproductive tissues and impair the function of the hypothalamic-pituitary-gonadal (HPG) axis, leading to disruptions in the secretion of key reproductive hormones. Consequently, the combined effects of oxidative stress and hormonal imbalances can significantly interfere with fertility, reducing both the quality of gametes and the overall reproductive health of individuals [60].

Excessive alcohol and caffeine consumption can impair fertility by disrupting hormone levels and increasing oxidative stress. Chronic alcohol use before pregnancy causes epigenetic changes in reproductive cells, leading to fertility issues. In males, alcohol can result in DNA hypomethylation in spermatogenesis genes like DNMT3a and DNMT3b, reducing sperm count and motility. It also impairs histone modifications, affecting sperm chromatin structure and fertilization ability. In females, alcohol alters DNA methylation in oocytes, particularly in genes like Bcl-2, crucial for oocyte survival, leading to irregular cycles, decreased ovarian reserve, and reduced fertility [61].

Additionally, alcohol exposure during pregnancy can cause specific epigenetic changes that affect the developing fetus and can be passed down through subsequent generations. Specifically, ethanol exposure can alter ncRNAs: miR-9, miR-21, miR-153, and miR-335. These are critical in regulating gene expression during neural development and cellular differentiation [62,63]. The altered expression of these miRNAs caused by maternal alcohol consumption can disrupt fetal brain development, resulting in neurodevelopmental disorders and congenital abnormalities. Furthermore, prenatal alcohol consumption can cause hypermethylation of the H19 gene and hypomethylation of the IGF2 gene, which disrupts fetal growth and development. Histone modifications, such as increased methylation at H3K27 and decreased acetylation at H3K9, can affect genes involved in the development of the HPG axis, such as Gonadotropin-Releasing Hormone 1 (GnRH), resulting in delayed puberty and reproductive dysfunction in offspring [63]. These changes can last into adulthood and be passed down to future generations via the germline, resulting in altered gene expression patterns, increased susceptibility to developmental and reproductive disorders, and potential long-term health consequences for offspring.

### 3.3. Lifestyle Factors

Physical activity (PA), stress, and sleep quality all have a significant impact on fertility via different physiological and hormonal mechanisms. Moderate physical activity improves fertility by increasing blood flow to reproductive organs and keeping a healthy weight, which is essential for hormonal balance. Exercise raises the level of sex hormone-binding globulin (SHBG), which controls free testosterone and estrogen levels. A recent systematic review and meta-analysis by Zhao et al. showed that excessive PA was inversely associated with fertility in the study population compared to the moderate PA [64]. The mechanism by which excessive exercise might impair fertility might be by raising cortisol levels and lowering GnRH, resulting in less LH and FSH secretion, impairing ovulation in women and spermatogenesis in men. Physical activity induces DNA hypomethylation in genes like PGC-1α, enhancing mitochondrial biogenesis and fatty acid oxidation, while also promoting histone modifications such as increased H3K4 methylation and H3K27 acetylation, which activate transcription of exercise-responsive genes. Exercise also upregulates beneficial miRNAs, such as miR-1 and miR-133a, that aid in muscle adaptation and differentiation. Conversely, physical inactivity leads to hypermethylation of genes like PGC-1α, increasing insulin resistance and disrupting metabolic health. These epigenetic changes collectively enhance or impair metabolic efficiency, oxidative capacity, and overall muscle function depending on the presence or absence of regular exercise [65]. These conditions increase inflammation and androgen production, impairing sperm and egg quality and resulting in irregular menstrual cycles, anovulation, and reduced fertility.

On the other hand, stress induces specific epigenetic changes that significantly impact gene expression and long-term health outcomes. Increased DNA methylation at the glucocorticoid receptor gene (NR3C1) promoter reduces its expression, impairing the HPA axis and prolonging stress responses. Prenatal stress causes hypomethylation of the CRF gene, increasing stress sensitivity in offspring. Stress also reduces histone acetylation at BDNF gene promoters, decreasing BDNF expression vital for neural plasticity. These epigenetic changes, including stress-induced methylation patterns in genes like MECP2 and CRFR2, can be inherited, affecting offspring behavior. Early-life stress increases DNA methylation in stress-related genes, while high maternal care decreases methylation and increases histone acetylation, promoting resilience [66]. Such stress-induced epigenetic changes can negatively impact fertility by disrupting hormonal balance, impairing reproductive function, and affecting the quality of gametes and the reproductive environment. Physiologically, chronic stress activates the HPA axis, which boosts cortisol and adrenaline production. Elevated cortisol disrupts the HPG axis, preventing the pulsatile release of GnRH and subsequent secretion of LH and FSH, which are required for ovulation and sperm production. A recent study investigated the impact of stress levels on infertility in women and found that those with elevated salivary alpha-amylase—a biomarker for stress—exhibited a 29% reduction in fertility and a twofold increase in the likelihood of infertility compared to women with lower stress levels [67].

According to European Society of Human Reproduction and Embryology (ESHRE) guidelines, comprehensive support is crucial in the treatment of infertility because standard treatments only help patients become parents; this results in emotional distress for most patients (23% of whom quit early), and about one-third of patients do not achieve success. This underscores the need for high-quality care that addresses both medical and psychosocial aspects of infertility [68].

Stress-induced oxidative stress damages oocyte and sperm DNA, reducing fertility. Nowak et al. have reviewed how prenatal stress triggers lasting epigenetic changes in offspring. Maternal stress during pregnancy alters DNA methylation in offspring, particularly in HPA axis-related genes like NR3C1 and FKBP51, leading to heightened cortisol responses and impaired stress regulation. Additionally, stress affects methylation of imprinted genes like IGF2, influencing growth and immune function, as well as socioemotional regulation genes like SLC6A4 and OXTR, potentially impacting long-term mental health and development [69].

Sleep quality significantly influences hormonal regulation through circadian rhythms. Sleep deprivation can lead to abnormal levels of key hormones essential for normal reproductive function, such as testosterone, thyroid-stimulating hormone, and luteinizing hormone. Disrupted hormone levels can adversely affect the menstrual cycle and ovulation in females, as well as reduce sperm quality in males [70]. Tang et al. discovered that sleep deprivation and poor sleep quality affect DNA methylation in regions regulating voltage-gated potassium channels, which are critical for cellular homeostasis and hormone balance. Sleep deprivation also causes aberrant DNA methylation in genes associated with reproductive health, altering hormone production and resulting in anovulation and irregular cycles. Furthermore, inadequate sleep reduces histone acetylation of H3 and H4, affecting chromatin structure and gene expression [23].

## 4. Epigenetics and Assisted Reproductive Technologies (ART)

ART laboratory procedures, such as sperm preparation and cryopreservation, have been found to impact the epigenetic landscape of gametes and embryos, with major consequences for both patient and offspring health. For example, sperm preparation methods, such as density gradient centrifugation and swim-up techniques, might alter the DNA methylation patterns of spermatozoa, particularly in genes involved in critical developmental processes. These changes could potentially impact embryonic development and the long-term health of the child [71]. Similarly, cryopreservation, which is routinely employed to preserve embryos and gametes, has been shown to influence their epigenetic stability. Cryopreservation can alter DNA methylation patterns in placental tissues, affecting gene expression and genomic integrity [71,72]. Specific ART procedures, such as ovarian stimulation and embryo culture, have also been associated with epigenetic changes, including abnormal DNA methylation at imprinting areas [73]. Research employing genome-wide techniques has found that ART is related with global hypomethylation, notably in repetitive sequences like LINE1. This may have long-term ramifications for the health and development of ART-conceived offspring [72]. Epigenetic modifications can impact both immediate outcomes, such as embryo viability and implantation success, and long-term health consequences for children conceived via ART [71,72].

Fauque et al. have investigated the link between female infertility, particularly endometriosis, and the risk of imprinting-related disorders in offspring conceived through ART [74]. Their study discovered that children born after fresh embryo transfers had a higher prevalence of imprinting-related diseases, such as neonatal diabetes mellitus, particularly if the mother had endometriosis, implying that both ART procedures and maternal conditions can cause epigenetic changes in the offspring. This study, along with others, emphasizes the importance of epigenetic modifications, specifically DNA methylation, in female infertility. Indeed, in their research, differential methylation profiles in endometrial tissue of women with endometriosis were identified, involving genes like HOXA10, PR, and ESR1, which are crucial for normal reproductive function [74]. The HOXA10 gene, which is essential for endometrial receptivity and implantation, was discovered to be hypermethylated in endometriosis patients. This hypermethylation occurs specifically in the promoter region of the HOXA10 gene, resulting in its reduced expression, negatively impacting the endometrial environment and contributing to infertility.

Moreover, altered DNA methylation patterns were found in sperm cells, leading to abnormalities in fetuses conceived through ART [73]. Song et al. have investigated DNA methylation differences between in vitro- and in vivo-conceived children, highlighting that the observed methylation changes are primarily associated with ART procedures rather than the underlying infertility. Their study showed that significant methylation differences were found at 37 CpG sites in 16 genes, which included differential methylation at CpG sites in genes like H19 and MEST [73].

### Transgenerational Inheritance, Epigenetic Reprogramming, and Genomic Imprinting

Recent studies have highlighted the importance of epigenetic profile inheritance across generations, especially in the context of ART. Transgenerational inheritance is the transmission of epigenetic markers, such as DNA methylation, from one generation to the next without changing the DNA sequence itself. This behavior is especially concerning in ART, as epigenetic alterations caused by laboratory methods may be transmitted by following generations, hence compounding the effects over time [72]. For example, aberrant DNA methylation at imprinted loci, such as the H19/IGF2 region, has been observed in ART-conceived children. These alterations are particularly problematic because they can lead to imprinting disorders, which are associated with various developmental and metabolic abnormalities [73].

Epigenetic reprogramming, the process of resetting epigenetic marks during gametogenesis and early embryonic development, is especially vulnerable to disruption by ART. Normally, this reprogramming ensures that embryos develop with the correct epigenetic instructions; however, ART treatments such as ovarian stimulation, embryo culture, and cryopreservation have been demonstrated to disrupt this process. The result is the persistence of abnormal epigenetic marks, which can lead to improper gene expression and increase the risk of imprinting disorders like Beckwith–Wiedemann syndrome and Angelman syndrome, which are more common in ART-conceived individuals [74].

These considerations underscore the importance of paying closer attention to the possibility of epigenetic modifications being passed down to future generations. As ART remains a popular treatment for infertility, it is critical to evaluate not only the immediate outcomes but also the long-term epigenetic health of subsequent generations. Research should focus on understanding how epigenetic alterations are preserved and inherited, and identifying techniques to reduce their effects, ensuring that the advantages of ART do not come with unexpected long-term consequences [72].

## 5. Diagnostic and Therapeutic Implications

Since epigenetic changes play a significant role in infertility, they have the potential to offer valuable opportunities for advancements in both diagnostic and therapeutic approaches. This section explores the current diagnostic methods for detecting epigenetic alterations and the potential therapeutic strategies targeting these epigenetic mechanisms to improve reproductive outcomes.

### 5.1. Current Diagnostic Methods for Detecting Epigenetic Changes

Methods for detecting epigenetic changes involve analyzing DNA methylation patterns, histone modifications, non-coding RNA expression, and chromatin structure alterations through various molecular and computational techniques (Table 4). Employing a combination of these methods can provide a more comprehensive understanding of epigenetic modifications. While bisulfite sequencing is widely regarded as the gold standard for DNA methylation analysis, due to its high accuracy and resolution, no single gold standard exists for all types of epigenetic modifications.

All these diagnostic approaches have drawbacks. While bisulfite sequencing gives high-resolution data on DNA methylation, it is time-consuming and costly, rendering it unsuitable for large-scale studies. Furthermore, the investigation of histone modifications and non-coding RNA expression frequently necessitates highly specialized equipment and expertise, making them inaccessible to many laboratories. These challenges highlight the need for more streamlined, cost-effective, and accessible diagnostic technologies that may be extensively used in clinical settings. Furthermore, while current approaches can detect epigenetic changes, they do not necessarily provide clear insights into the functional repercussions of these changes, which is crucial for converting discoveries into therapeutic applications.

The method used should be determined by the specific research question and the type of epigenetic change under investigation. Combining multiple methods is generally recommended to compensate for the individual limitations of each method and to achieve a more robust and comprehensive understanding of the epigenetic landscape.

### 5.2. Potential Therapeutic Strategies Targeting Epigenetic Mechanisms

Although epigenetic drugs like DNA methylation inhibitors, histone deacetylase inhibitors, and non-coding RNA therapies show promise for treating infertility by targeting abnormal epigenetic modifications, their use in fertility treatment is complicated by significant challenges. These drugs are not specifically targeted to reproductive tissues, often leading to severe side effects, such as toxicity and off-target effects. Moreover, the methylation patterns they affect are not exclusive to infertility, impacting multiple genes and pathways in various tissues, which complicates their use solely for fertility treatments. This lack of specificity, combined with the potential for widespread genomic disruption, underscores the need for more targeted and safer therapeutic strategies.

Table 5 summarizes various epigenetic therapies currently utilized, including their mechanisms of action, in the treatment of different medical conditions. For example, DNA methylation inhibitors such as 5-azacytidine and Decitabine are used to treat myelodysplastic syndromes and acute myeloid leukemia, despite the risks of hematologic toxicity and immunosuppression [81]. Vorinostat and Romidepsin are histone deacetylase inhibitors that target cutaneous and peripheral T-cell lymphoma, respectively, but their use can cause fatigue, nausea, and thrombocytopenia [82]. Tazemetostat, a histone methyltransferase inhibitor, is used to treat follicular lymphoma and epithelioid sarcoma [83]. Possible side effects include nausea, vomiting, and anemia. BET inhibitors, such as JQ1 and OTX015, are used to treat NUT midline carcinoma and hematologic malignancies, but they can cause gastrointestinal issues and fatigue [84].

Through controlling gene expression after transcription, non-coding RNA therapies like siRNAs and miRNAs have demonstrated promise in treating a range of genetic disorders and cancers [85]. Since miRNA mimics and antimiRs are engineered to specifically target genes involved in reproductive processes, they hold great promise as therapeutic agents for more targeted interventions. While antimiRs can suppress overactive miRNAs, miRNA mimics can replace missing miRNAs. Even with these developments, issues with tolerability, delivery, and specificity still exist. For instance, in clinical trials, the miR-34 mimic, MRX34, resulted in notable adverse reactions, such as cytokine release syndrome, underscoring the significance of enhanced delivery strategies and meticulous toxicity evaluations. However, there have been no serious side effects from miR-16 restitution therapy or the intradermal injection of miR-29 mimic relapse, suggesting that miRNAs can be developed into safe therapeutic alternatives. These therapeutic approaches, while promising, emphasize the need for more focused and secure methods to lower side effects and boost treatment effectiveness [85].

To summarize, while epigenetic therapies have considerable potential for treating infertility, it is equally necessary to critically evaluate their limitations and problems. The development of more precise and tissue-specific therapy techniques is critical to reducing hazards and increasing treatment success. This necessitates continued study to better understand the intricate interplay between epigenetic changes and infertility, as well as advancements in medication delivery technologies that can provide more focused therapies.

## 6. Challenges and Future Directions

Despite significant advances in understanding epigenetic mechanisms in fertility, several important knowledge gaps remain. These include the precise roles of specific epigenetic modifications in gametogenesis and early embryonic development, the long-term effects of epigenetic therapies, and the influence of environmental factors on epigenetic marks. Furthermore, comprehensive research is required to better understand the interaction of genetic and epigenetic factors in infertility. Future research should elucidate the detailed mechanisms of epigenetic regulation in reproductive processes, develop more targeted and specific epigenetic therapies with fewer side effects, and investigate the molecular basis of infertility. Large-scale longitudinal studies should also be prioritized to better understand the impact of environmental and lifestyle factors on fertility-related epigenetic changes. The integration of epigenetic data and personalized medicine has enormous potential for improving fertility treatments. Personalized approaches could involve tailoring epigenetic therapies to an individual’s unique epigenetic profile, resulting in more effective and targeted interventions. Advances in genomics and bioinformatics will enable the identification of distinct epigenetic signatures associated with infertility, paving the way for personalized therapeutic strategies that optimize reproductive outcomes for each individual.

Restoring spermatogenesis, the process of sperm production, is relatively more feasible because sperm is continuously produced throughout a man’s life, with a new cycle completing every 64–72 days. This ongoing production allows for repeated opportunities to correct or improve the process through therapeutic interventions. The continual nature of spermatogenesis allows for continued cellular turnover, with damaged or epigenetically changed sperm potentially being replaced by healthier sperm after treatment. For example, therapies that target DNA methylation mistakes or histone changes in spermatogenic cells have the potential to increase sperm quality over time. Because of this plasticity in spermatogenesis, therapies can be repeated and altered based on the individual’s reaction, increasing the possibility of restoring normal fertility. In contrast, oogenesis, the development of mature egg cells in females, is more complex and occurs during fetal development, with a finite number of oocytes established before birth. This finite nature of oogenesis means that any epigenetic alterations that occur, whether due to environmental factors or genetic predispositions, can have long-lasting impacts on a woman’s fertility. For example, epigenetic changes in oocytes, such as abnormal DNA methylation or histone acetylation, are difficult to reverse or rectify since the pool of viable oocytes is not as regenerative as sperm. Furthermore, oogenesis is regulated by maternal lifestyle and environmental factors throughout pregnancy. These factors can influence the epigenetic control of developing oocytes, potentially affecting the offspring’s future fertility. For example, exposure to environmental toxins, poor nutrition, or stress during pregnancy can lead to epigenetic changes in the developing oocytes, which may compromise the reproductive potential of the female offspring. In this way, the epigenetic environment during fetal development plays a critical role in determining the quality and viability of a woman’s oocytes later in life. The distinction between spermatogenesis and oogenesis underscores the inherent difficulties in treating female infertility associated to oogenesis as opposed to male infertility. Because sperm production is continuous, treatments aimed at male infertility that restore spermatogenesis are more likely to be successful. However, preventative approaches, such as promoting healthy maternal lifestyle practices during pregnancy, may help optimize the epigenetic landscape of developing oocytes, potentially improving the reproductive health of future generations. Ultimately, while there are clear differences in the feasibility of treating male versus female infertility, due to the nature of spermatogenesis and oogenesis, there are also shared challenges in addressing epigenetic alterations that affect reproductive health. Both sexes may benefit from advancements in epigenetic therapies, though the strategies and expected outcomes will differ significantly due to the biological differences in how male and female gametes are produced and maintained.

Recent advances in epigenetic therapies provide promising strategies for treating infertility. One of the most significant developments has been the use of mesenchymal stem cells (MSCs), which have shown promise in restoring fertility via epigenetic reprogramming. MSCs can differentiate into a variety of cell types, including germ-like cells, and have been used to treat conditions like POI and other reproductive issues. These cells can alter DNA methylation and histone modification patterns, enhancing the regenerative capacity of reproductive tissues [86,87]. However, translating MSC-based therapies into clinical practice has considerable hurdles, including the danger of unintentional epigenetic changes that could result in abnormal gene expression in non-target tissues. Furthermore, the long-term implications of MSC therapy, particularly the potential for epigenetic modifications to remain across generations, must be thoroughly investigated before these therapies are widely implemented. Additionally, mitochondrial nutrient therapy has emerged as a novel approach to combating infertility caused by mitochondrial dysfunction. Supplements such as resveratrol (RSV) can improve mitochondrial function and reduce oxidative stress, which is essential for preserving oocyte quality and delaying ovarian aging. In detail, RSV inhibits the activity of DNMTs, specifically DNMT1, DNMT3a, and DNMT3b, resulting in the hypomethylation of gene promoters such as BRCA1, p53, and p21. Hypomethylation can reactivate tumor suppressor genes and enhance cellular function. RSV also affects histone modifications by decreasing repressive marks, like H3K27me3 and H4R3me2s, and increasing activating marks, like H3K9ac and H3K27ac, which promote a more open chromatin structure and increase gene expression. This epigenetic modification is essential for activating genes involved in mitochondrial biogenesis and the oxidative stress response [88,89]. Despite these benefits, the non-specific nature of RSV’s epigenetic effects raises concerns about potential off-target effects, including the reactivation of oncogenes or the disruption of other critical pathways not related to fertility. RSV also affects the expression of miR-338-3p and miR-92b-3p, which are involved in osteogenic differentiation and inflammation [88,89]. Lastly, its most important activity, activation of SIRT1, enhances mitochondrial function and biogenesis. This is primarily achieved through the deacetylation and activation of peroxisome proliferator-activated receptor gamma coactivator 1-alpha (PGC-1α), a key regulator of mitochondrial biogenesis and energy metabolism [88]. SIRT1 activation promotes the expression of genes involved in mitochondrial respiration and biogenesis, thereby preserving mitochondrial integrity and function. This is critical for cells with high energy demands, such as oocytes and sperm, and thus supports fertility. Since SIRT1 promotes the recruitment of primordial follicles, all natural compounds that affects its activation can be used for in vitro activation (IVA) procedures [90]. IVA is a novel technique for stimulating dormant ovarian follicles to develop and mature. It is especially beneficial for women with primary ovarian insufficiency. IVA activates follicles by utilizing our understanding of signaling pathways and epigenetic mechanisms, which improves the results of fertility [91]. However, the use of IVA raises ethical concerns, notably about the possibilities of generating unintended genetic or epigenetic modifications that could impair the offspring’s long-term health. The requirement for precise control over the activation process, as well as the possibility of off-target consequences, highlights the importance of comprehensive preclinical and clinical research before incorporating these therapies safely and effectively into routine infertility treatments.

These novel approaches, which are still being investigated, highlight the potential of epigenetic therapies to revolutionize fertility treatment by addressing the underlying epigenetic abnormalities that contribute to infertility. Additional research and clinical trials will be necessary to refine these therapies and ensure their safety and efficacy for widespread use.

## 7. Conclusions

In summary, infertility results from a complex interplay of genetic, environmental, and lifestyle factors, with recent research highlighting the crucial role of epigenetic mechanisms. DNA methylation, histone modification, and non-coding RNAs play pivotal roles in regulating fertility through their impact on gene expression during gametogenesis, affecting both spermatogenesis and oogenesis. Environmental factors such as diet, stress, and exposure to toxins can induce epigenetic changes, potentially disrupting normal reproductive processes and leading to infertility. The influence of stress on epigenetic modifications highlights the importance of incorporating psychological counseling into infertility treatment. Stress can cause epigenetic modifications that negatively impact reproductive health and, in turn, impact the efficacy of infertility treatments. By managing stress, psychological counseling may help mitigate its deleterious epigenetic effects. Looking ahead, personalized medicine holds the key to the future of infertility treatment, as understanding and manipulating the epigenetic mechanisms underlying reproductive health will enable tailored therapies that address individuals’ specific needs. This personalized approach is anticipated to enhance the efficacy of infertility treatments and lead to better patient outcomes.

## Figures and Tables

**Table 1:** Impaired DNA Methylation in Male Fertility.

Gene/ Region	Gene Function	Position in Gene	Description
Hypomethylation			
H19	A long non-coding RNA involved in growth control and development, acting as a tumor suppressor	CpG 1, 3, 6 within 220-bp Fragment (chromosome 11)—located near the promoter region but can extend into the gene body	Decreased methylation in the H19 gene linked to idiopathic male infertility. Lower methylation rates found in infertile males compared to controls, indicating a correlation with male infertility.
IGF2-H19	Involved in growth and development.	Promoter region	Hypomethylation in this locus is associated with abnormalities in fetuses conceived through assisted reproductive techniques.
DAZL	An RNA-binding protein essential for gametogenesis, specifically in the development of sperm	Promoter region	Reduced methylation in the promoter region resulting in impaired spermatogenesis and sperm dysfunction.
Hypermethylation			
MTHFR	Plays a critical role in DNA and folate synthesis, as well as in the DNA methylation process itself.	Promoter region	Abnormal methylation leads to reduced enzyme activity, reduced methionine availability and impaired DNA methylation
MEST	Involved in cell differentiation and growth, particularly in mesodermal tissues, and imprinting control	Promoter region	Increased methylation in the promoter region causing defective sperm production and oligozoospermia.
SNRPN	Involved in RNA processing and is an important component of the Prader–Willi syndrome region	Promoter region	Increased methylation in the promoter region associated with impaired spermatogenesis.
GNAS	Involved in cell differentiation and growth, particularly in mesodermal tissues, and imprinting control.	32 CpG sites within 343-bp fragment (chromosome 20)—includes regions around the promoter but can also encompass regulatory elements within the gene	Significant aberrant DNA methylation found in 21.5% of infertile men at the GNAS locus.
DIRAS3	involved in growth regulation and development via parent-of-origin expression.	13 CpG sites within 207-bp fragment (chromosome 1)—encompassing promoter regions and nearby regulatory elements.	Significant aberrant DNA methylation found in 22.2% of infertile men at the DIRAS3 locus.

**Table 2:** Impaired DNA Methylation in Female Fertility.

Gene/Region	Gene Function	Position in Gene	Description
Hypomethylation			
CYP19A1	Encodes aromatase, key enzyme in estrogen biosynthesis	Promoter region	Methylation changes affect estrogen biosynthesis, contributing to PCOS
FKBP5	Encodes a protein that binds to immunophilin and glucocorticoid receptors	Promoter region	Altered methylation impacts stress response and glucocorticoid signaling, contributing to PCOS
YAP1	Transcriptional regulator involved in cell proliferation and apoptosis	Promoter region	Changes in methylation affect cell proliferation and apoptosis, associated with PCOS
KDM6A	Histone demethylase involved in chromatin modification	Promoter region	Hypomethylation affects chromatin structure and gene expression, related to Turner syndrome
LHCGR	Encodes the receptor for luteinizing hormone/choriogonadotropin	Promoter region	Methylation changes impact hormone receptor expression, associated with PCOS
USP9X	Deubiquitinating enzyme involved in protein degradation	Promoter region	Hypomethylation impacts protein degradation pathways, related to Turner syndrome
ZFX	Transcription factor involved in cell differentiation and proliferation	Promoter region	Hypomethylation affects transcription regulation, related to Turner syndrome
Hypermethylation			
HOXA10	Homeobox gene involved in endometrial receptivity	Promoter region	Altered methylation affects endometrial receptivity, contributing to endometriosis
PR	Progesterone receptor involved in female reproductive tissue development	Promoter	Hypermethylation of the PR promoter region is associated with reduced gene expression, which can impair reproductive tissue development and function.
ESR1	Estrogen receptor involved in hormone signaling and reproductive tissue regulation	Promoter	Hypermethylation of the ESR1 promoter region leads to decreased expression of estrogen receptors, potentially affecting hormone signaling and reproductive tissue health

**Table 3:** Impact of Heavy Metals on Fertility.

Heavy Metal	Negative Impact on Fertility	Epigenetic Changes
Arsenic	Triggers DNA methylation, deregulates spermatogenesis, and decreases sperm quality	Increased H3K9me3 in testes, suppression of steroidogenic genes (Star, P450scc, Hsd3b, Cyp17a1, Hsd17b), reduced histone H3K9me2/3 in Leydig cells, increased Hsd3b expression, and disrupted steroid hormone biosynthesis
Cadmium	Lowers testosterone levels, reduces sperm quality, and affects Sertoli and Leydig cells	Global DNA methylation increase, hypermethylation of Mdr1b gene promoter in BTB, hypomethylation of LINE-1 in testes, increased H3K9me1/2 methylation, and apoptosis
Lead	Disrupts endocrine function, causes oxidative stress, lowers LH and FSH secretion, causes irregular menstrual cycles, and impaired spermatogenesis	Sex-dependent and gene-specific DNA methylation differences in DMRs of PEG3, IGF2/H19, and PLAGL1/HYMAI in adulthood
Mercury	Accumulates in reproductive organs, produces ROS, increases oxidative stress, and causes DNA damage to oocytes and sperm cells	Changes in DNA methylation at CpG sites cg12204245 (GRK1), cg02212000 (GGH), and cg24184221 (MED31); impacts lipid metabolism and RNA polymerase II transcription

**Table 4:** Diagnostic Methods for Detecting Epigenetic Changes.

Method	Description	Advantages	Limitations	Ref.
Bisulfite Sequencing	Converts unmethylated cytosines to uracil, then is sequenced to detect methylated cytosines	High resolution, quantitative, genome-wide analysis	Time-consuming, requires large amounts of DNA	[75]
Chromatin Immunoprecipitation (ChIP)	Uses antibodies to isolate DNA-protein complexes, followed by sequencing (ChIP-seq)	Identifies specific protein–DNA interactions and histone modifications	Requires high-quality antibodies and can be technically challenging	[76]
Methylation-Specific PCR (MSP)	Amplifies DNA regions, differentiating between methylated and unmethylated sequences	Simple, cost-effective, and quick results	Limited to known sequences and not quantitative	[77]
Microarray Analysis	Uses probes to detect methylation changes across the genome	High throughput, covers large genomic regions	Lower resolution than sequencing and has potential for hybridization errors	[78]
Next-Generation Sequencing (NGS)	Provides comprehensive profiling of epigenetic marks at high resolution and throughput	High resolution, quantitative, and genome-wide	Expensive and requires extensive data analysis	[79]
RNA Sequencing (RNA-seq)	Measures non-coding RNA levels to infer epigenetic regulatory changes	High resolution, identifies novel transcripts	Requires high-quality RNA and involves complex data analysis	[80]

**Table 5:** Drugs That Target Epigenetic Modifications.

Category	Mechanisms of Action	Examples of Drugs	Disease/Condition	Ref.
DNA Methylation Inhibitors	Inhibit DNA methyltransferases (DNMTs) to reactivate silenced genes	5-azacytidine, Decitabine	Myelodysplastic syndromes, Acute myeloid leukemia	[81]
Histone Deacetylase Inhibitors (HDACi)	Inhibit HDACs to promote histone acetylation and gene expression	Vorinostat, Romidepsin	Cutaneous T-cell lymphoma, Peripheral T-cell lymphoma	[82]
Histone Methyltransferase Inhibitors	Inhibit EZH2 to alter histone methylation and reactivate tumor suppressor genes	Tazemetostat (EZH2 inhibitor)	Follicular lymphoma, Epithelioid sarcoma	[83]
Bromodomain and Extra-Terminal Domain (BET) Inhibitors	Disrupt BET protein binding to acetylated histones, affecting transcription	JQ1, OTX015	NUT midline carcinoma, Hematologic malignancies	[84]
Non-coding RNA Therapies	Regulate gene expression post-transcriptionally	miRNAs, siRNAs	Various cancers, Genetic disorders	[85]

## Data Availability

No new data was created or analyzed in this study. Data sharing is not applicable to this article.

## References

[1] Vander Borght M., Wyns C. (2018). Fertility and Infertility: Definition and Epidemiology. Clin. Biochem..

[2] Hoffman B.L., Schorge J.O., Bradshaw K.D., Halvorson L.M., Schaffer J.I., Corton M.M. (2016). Evaluation of the Infertile Couple. Williams Gynecology.

[3] Das L., Parbin S., Pradhan N., Kausar C., Patra S.K. (2017). Epigenetics of Reproductive Infertility. Front. Biosci. Schol. Ed..

[4] Liu L., Li Y., Tollefsbol T.O. (2008). Gene-Environment Interactions and Epigenetic Basis of Human Diseases. Curr. Issues Mol. Biol..

[5] Walker M.H., Tobler K.J. (2024). Female Infertility. StatPearls.

[6] Rotondo J.C., Lanzillotti C., Mazziotta C., Tognon M., Martini F. (2021). Epigenetics of Male Infertility: The Role of DNA Methylation. Front. Cell Dev. Biol..

[7] Guzick D.S., Overstreet J.W., Factor-Litvak P., Brazil C.K., Nakajima S.T., Coutifaris C., Carson S.A., Cisneros P., Steinkampf M.P., Hill J.A. (2001). Sperm Morphology, Motility, and Concentration in Fertile and Infertile Men. N. Engl. J. Med..

[8] Pisarska M.D., Chan J.L., Lawrenson K., Gonzalez T.L., Wang E.T. (2019). Genetics and Epigenetics of Infertility and Treatments on Outcomes. J. Clin. Endocrinol. Metab..

[9] Moore L.D., Le T., Fan G. (2013). DNA Methylation and Its Basic Function. Neuropsychopharmacology.

[10] Ludwig A.K., Zhang P., Cardoso M.C. (2016). Modifiers and Readers of DNA Modifications and Their Impact on Genome Structure, Expression, and Stability in Disease. Front. Genet..

[11] Holliday R. (1989). DNA Methylation and Epigenetic Mechanisms. Cell Biophys..

[12] Guerrero-Bosagna C., Skinner M.K. (2014). Environmentally Induced Epigenetic Transgenerational Inheritance of Male Infertility. Curr. Opin. Genet. Dev..

[13] Botezatu A., Socolov R., Socolov D., Iancu I.V., Anton G. (2014). Methylation Pattern of Methylene Tetrahydrofolate Reductase and Small Nuclear Ribonucleoprotein Polypeptide N Promoters in Oligoasthenospermia: A Case-Control Study. Reprod. Biomed. Online.

[14] Leclerc D., Sibani S., Rozen R. (2013). Molecular Biology of Methylenetetrahydrofolate Reductase (MTHFR) and Overview of Mutations/Polymorphisms. Madame Curie Bioscience Database.

[15] Shacfe G., Turko R., Syed H.H., Masoud I., Tahmaz Y., Samhan L.M., Alkattan K., Shafqat A., Yaqinuddin A. (2023). A DNA Methylation Perspective on Infertility. Genes.

[16] Tang Q., Pan F., Yang J., Fu Z., Lu Y., Wu X., Han X., Chen M., Lu C., Xia Y. (2018). Idiopathic Male Infertility Is Strongly Associated with Aberrant DNA Methylation of Imprinted Loci in Sperm: A Case-Control Study. Clin. Epigenet..

[17] Li X.-P., Hao C.-L., Wang Q., Yi X.-M., Jiang Z.-S. (2016). H19 Gene Methylation Status Is Associated with Male Infertility. Exp. Ther. Med..

[18] Urdinguio R.G., Bayón G.F., Dmitrijeva M., Toraño E.G., Bravo C., Fraga M.F., Bassas L., Larriba S., Fernández A.F. (2015). Aberrant DNA Methylation Patterns of Spermatozoa in Men with Unexplained Infertility. Hum. Reprod..

[19] Navarro-Costa P., Nogueira P., Carvalho M., Leal F., Cordeiro I., Calhaz-Jorge C., Gonçalves J., Plancha C.E. (2010). Incorrect DNA Methylation of the DAZL Promoter CpG Island Associates with Defective Human Sperm. Hum. Reprod..

[20] Boissonnas C.C., Abdalaoui H.E., Haelewyn V., Fauque P., Dupont J.M., Gut I., Vaiman D., Jouannet P., Tost J., Jammes H. (2010). Specific Epigenetic Alterations of IGF2-H19 Locus in Spermatozoa from Infertile Men. Eur. J. Hum. Genet..

[21] Uysal F., Ozturk S. (2020). The Loss of Global DNA Methylation Due to Decreased DNMT Expression in the Postnatal Mouse Ovaries May Associate with Infertility Emerging during Ovarian Aging. Histochem. Cell Biol..

[22] Barišić A., Kolak M., Peterlin A., Tul N., Gašparović Krpina M., Ostojić S., Peterlin B., Pereza N. (2020). DNMT3B Rs1569686 and Rs2424913 Gene Polymorphisms Are Associated with Positive Family History of Preterm Birth and Smoking Status. Croat. Med. J..

[23] Tang Y., Gan H., Wang B., Wang X., Li M., Yang Q., Geng M., Zhu P., Shao S., Tao F. (2023). Mediating Effects of DNA Methylation in the Association between Sleep Quality and Infertility among Women of Childbearing Age. BMC Public Health.

[24] Bannister A.J., Kouzarides T. (2011). Regulation of Chromatin by Histone Modifications. Cell Res..

[25] Wang T., Gao H., Li W., Liu C. (2019). Essential Role of Histone Replacement and Modifications in Male Fertility. Front. Genet..

[26] Oikawa M., Simeone A., Hormanseder E., Teperek M., Gaggioli V., O’Doherty A., Falk E., Sporniak M., D’Santos C., Franklin V.N.R. (2020). Epigenetic Homogeneity in Histone Methylation Underlies Sperm Programming for Embryonic Transcription. Nat. Commun..

[27] Hammoud S.S., Nix D.A., Hammoud A.O., Gibson M., Cairns B.R., Carrell D.T. (2011). Genome-Wide Analysis Identifies Changes in Histone Retention and Epigenetic Modifications at Developmental and Imprinted Gene Loci in the Sperm of Infertile Men. Hum. Reprod..

[28] Okada Y., Tateishi K., Zhang Y. (2010). Histone Demethylase JHDM2A Is Involved in Male Infertility and Obesity. J. Androl..

[29] Navarro-Costa P., McCarthy A., Prudêncio P., Greer C., Guilgur L.G., Becker J.D., Secombe J., Rangan P., Martinho R.G. (2016). Early Programming of the Oocyte Epigenome Temporally Controls Late Prophase I Transcription and Chromatin Remodelling. Nat. Commun..

[30] Bogliotti Y.S., Ross P.J. (2012). Mechanisms of Histone H3 Lysine 27 Trimethylation Remodeling during Early Mammalian Development. Epigenetics.

[31] Cech T.R., Steitz J.A. (2014). The Noncoding RNA Revolution-Trashing Old Rules to Forge New Ones. Cell.

[32] Saliminejad K., Khorram Khorshid H.R., Soleymani Fard S., Ghaffari S.H. (2019). An Overview of microRNAs: Biology, Functions, Therapeutics, and Analysis Methods. J. Cell. Physiol..

[33] Xu W., Jiang X., Huang L. (2019). RNA Interference Technology. Compr. Biotechnol..

[34] Wei J.-W., Huang K., Yang C., Kang C.-S. (2017). Non-Coding RNAs as Regulators in Epigenetics (Review). Oncol. Rep..

[35] Wang C., Yang C., Chen X., Yao B., Yang C., Zhu C., Li L., Wang J., Li X., Shao Y. (2011). Altered Profile of Seminal Plasma microRNAs in the Molecular Diagnosis of Male Infertility. Clin. Chem..

[36] Sirotkin A.V., Ovcharenko D., Grossmann R., Lauková M., Mlyncek M. (2009). Identification of microRNAs Controlling Human Ovarian Cell Steroidogenesis via a Genome-Scale Screen. J. Cell. Physiol..

[37] Fu Q., Wang P.J. (2014). Mammalian piRNAs: Biogenesis, Function, and Mysteries. Spermatogenesis.

[38] Gu A., Ji G., Shi X., Long Y., Xia Y., Song L., Wang S., Wang X. (2010). Genetic Variants in Piwi-Interacting RNA Pathway Genes Confer Susceptibility to Spermatogenic Failure in a Chinese Population. Hum. Reprod..

[39] Zhu Q., Kirby J.A., Chu C., Gou L.-T. (2021). Small Noncoding RNAs in Reproduction and Infertility. Biomedicines.

[40] Chen X., Zheng Y., Lei A., Zhang H., Niu H., Li X., Zhang P., Liao M., Lv Y., Zhu Z. (2020). Early Cleavage of Preimplantation Embryos Is Regulated by tRNAGln-TTG–Derived Small RNAs Present in Mature Spermatozoa. J. Biol. Chem..

[41] Derakhshan Z., Bahmanpour S., Fallahi J., Tabei M.B., Tabei S.M.B. (2023). The Role of Circular RNAs in Male Infertility and Reproductive Cancers: A Narrative Review. Iran. J. Med. Sci..

[42] Loda A., Heard E. (2019). Xist RNA in Action: Past, Present, and Future. PLoS Genet..

[43] Dandolo L., Monnier P., Tost J. (2017). The *H19* Locus. Reference Module in Life Sciences.

[44] Peng Y., Guo R., Shi B., Li D. (2023). The Role of Long Non-Coding RNA H19 in Infertility. Cell Death Discov..

[45] Arun G., Aggarwal D., Spector D.L. (2020). MALAT1 Long Non-Coding RNA: Functional Implications. Noncod. RNA.

[46] Asl A.-J., Sharifi M., Dashti A., Dashti G.R. (2023). Relationship between Long Non-Coding RNA MALAT1 and HOTAIR Expression with Sperm Parameters, DNA and Malondialdehyde Levels in Male Infertility. Tissue Cell.

[47] Leygue E. (2007). Steroid Receptor RNA Activator (SRA1): Unusual Bifaceted Gene Products with Suspected Relevance to Breast Cancer. Nucl. Recept. Signal.

[48] Wang W., Wang R., Liu R. (2015). Progress in research on imprinted gene associated with male infertility. Zhonghua Yi Xue Yi Chuan Xue Za Zhi.

[49] Hamilton S., de Cabo R., Bernier M. (2020). Maternally Expressed Gene 3 in Metabolic Programming. Biochim. Biophys. Acta Gene Regul. Mech..

[50] Vessa B., Perlman B., McGovern P.G., Morelli S.S. (2022). Endocrine Disruptors and Female Fertility: A Review of Pesticide and Plasticizer Effects. F S Rep..

[51] Alavian-Ghavanini A., Rüegg J. (2018). Understanding Epigenetic Effects of Endocrine Disrupting Chemicals: From Mechanisms to Novel Test Methods. Basic Clin. Pharmacol. Toxicol..

[52] Li Y., Xie C., Murphy S.K., Skaar D., Nye M., Vidal A.C., Cecil K.M., Dietrich K.N., Puga A., Jirtle R.L. (2016). Lead Exposure during Early Human Development and DNA Methylation of Imprinted Gene Regulatory Elements in Adulthood. Environ. Health Perspect..

[53] Massányi P., Massányi M., Madeddu R., Stawarz R., Lukáč N. (2020). Effects of Cadmium, Lead, and Mercury on the Structure and Function of Reproductive Organs. Toxics.

[54] Lozano M., Yousefi P., Broberg K., Soler-Blasco R., Miyashita C., Pesce G., Kim W.J., Rahman M., Bakulski K.M., Haug L.S. (2022). DNA Methylation Changes Associated with Prenatal Mercury Exposure: A Meta-Analysis of Prospective Cohort Studies from PACE Consortium. Environ. Res..

[55] Ikokide E.J., Oyagbemi A.A., Oyeyemi M.O. (2022). Impacts of Cadmium on Male Fertility: Lessons Learnt so Far. Andrologia.

[56] Han X., Huang Q. (2021). Environmental Pollutants Exposure and Male Reproductive Toxicity: The Role of Epigenetic Modifications. Toxicology.

[57] Okpashi V.E., Ebunta A.F., Kumar N. (2021). Predicting the Outcome of Arsenic Toxicity on Exposed Juvenile Male-Humans: A Shift to Infertility. Arsenic Toxicity: Challenges and Solutions.

[58] Speckmann B., Grune T. (2015). Epigenetic Effects of Selenium and Their Implications for Health. Epigenetics.

[59] Mahmoud A.M. (2022). An Overview of Epigenetics in Obesity: The Role of Lifestyle and Therapeutic Interventions. Int. J. Mol. Sci..

[60] Cabler S., Agarwal A., Flint M., Du Plessis S.S. (2010). Obesity: Modern Man’s Fertility Nemesis. Asian J. Androl..

[61] Goldberg E.M., Aliani M. (2018). Metabolomics and Fetal Alcohol Spectrum Disorder. Biochem. Cell Biol..

[62] Balaraman S., Tingling J.D., Tsai P.-C., Miranda R.C. (2013). Dysregulation of microRNA Expression and Function Contributes to the Etiology of Fetal Alcohol Spectrum Disorders. Alcohol. Res..

[63] Akison L.K., Moritz K.M., Reid N. (2019). Adverse Reproductive Outcomes Associated with Fetal Alcohol Exposure: A Systematic Review. Reproduction.

[64] Zhao F., Hong X., Wang W., Wu J., Wang B. (2022). Effects of Physical Activity and Sleep Duration on Fertility: A Systematic Review and Meta-Analysis Based on Prospective Cohort Studies. Front. Public Health.

[65] Plaza-Diaz J., Izquierdo D., Torres-Martos Á., Baig A.T., Aguilera C.M., Ruiz-Ojeda F.J. (2022). Impact of Physical Activity and Exercise on the Epigenome in Skeletal Muscle and Effects on Systemic Metabolism. Biomedicines.

[66] Gudsnuk K., Champagne F.A. (2012). Epigenetic Influence of Stress and the Social Environment. ILAR J..

[67] Lynch C.D., Sundaram R., Maisog J.M., Sweeney A.M., Buck Louis G.M. (2014). Preconception Stress Increases the Risk of Infertility: Results from a Couple-Based Prospective Cohort Study—The LIFE Study. Hum. Reprod..

[68] Gameiro S., Boivin J., Dancet E., de Klerk C., Emery M., Lewis-Jones C., Thorn P., Van den Broeck U., Venetis C., Verhaak C.M. (2015). ESHRE Guideline: Routine Psychosocial Care in Infertility and Medically Assisted Reproduction-a Guide for Fertility Staff. Hum. Reprod..

[69] Nowak A.L., Anderson C.M., Mackos A.R., Neiman E., Gillespie S.L. (2020). Stress during Pregnancy and Epigenetic Modifications to Offspring DNA: A Systematic Review of Associations and Implications for Preterm Birth. J. Perinat. Neonatal Nurs..

[70] Michels K.A., Mendola P., Schliep K.C., Yeung E.H., Ye A., Dunietz G.L., Wactawski-Wende J., Kim K., Freeman J.R., Schisterman E.F. (2020). The Influences of Sleep Duration, Chronotype, and Nightwork on the Ovarian Cycle. Chronobiol. Int..

[71] Mani S., Ghosh J., Coutifaris C., Sapienza C., Mainigi M. (2020). Epigenetic Changes and Assisted Reproductive Technologies. Epigenetics.

[72] Håberg S.E., Page C.M., Lee Y., Nustad H.E., Magnus M.C., Haftorn K.L., Carlsen E.Ø., Denault W.R.P., Bohlin J., Jugessur A. (2022). DNA Methylation in Newborns Conceived by Assisted Reproductive Technology. Nat. Commun..

[73] Song S., Ghosh J., Mainigi M., Turan N., Weinerman R., Truongcao M., Coutifaris C., Sapienza C. (2015). DNA Methylation Differences between in Vitro- and in Vivo-Conceived Children Are Associated with ART Procedures Rather than Infertility. Clin. Epigenet..

[74] Fauque P., De Mouzon J., Devaux A., Epelboin S., Gervoise-Boyer M.-J., Levy R., Valentin M., Viot G., Bergère A., De Vienne C. (2020). Reproductive Technologies, Female Infertility, and the Risk of Imprinting-Related Disorders. Clin. Epigenet..

[75] Darst R.P., Pardo C.E., Ai L., Brown K.D., Kladde M.P. (2010). Bisulfite Sequencing of DNA. Curr. Protoc. Mol. Biol..

[76] Turner B. (2001). ChIP with Native Chromatin: Advantages and Problems Relative to Methods Using Cross-Linked Material. Mapping Protein/DNA Interactions by Cross-Linking.

[77] Cottrell S.E., Laird P.W. (2003). Sensitive Detection of DNA Methylation. Ann. N. Y Acad. Sci..

[78] Colyer H.A.A., Armstrong R.N., Mills K.I. (2012). Microarray for Epigenetic Changes: Gene Expression Arrays. Methods Mol. Biol..

[79] Meaburn E., Schulz R. (2012). Next Generation Sequencing in Epigenetics: Insights and Challenges. Semin. Cell Dev. Biol..

[80] Chen X., Xu H., Shu X., Song C.-X. (2023). Mapping Epigenetic Modifications by Sequencing Technologies. Cell Death Differ..

[81] Mei M., Aldoss I., Marcucci G., Pullarkat V. (2019). Hypomethylating Agents in Combination with Venetoclax for Acute Myeloid Leukemia: Update on Clinical Trial Data and Practical Considerations for Use. Am. J. Hematol..

[82] Yoon S., Eom G.H. (2016). HDAC and HDAC Inhibitor: From Cancer to Cardiovascular Diseases. Chonnam Med. J..

[83] Julia E., Salles G. (2021). EZH2 Inhibition by Tazemetostat: Mechanisms of Action, Safety and Efficacy in Relapsed/Refractory Follicular Lymphoma. Future Oncol..

[84] Riveiro M.E., Astorgues-Xerri L., Vazquez R., Frapolli R., Kwee I., Rinaldi A., Odore E., Rezai K., Bekradda M., Inghirami G. (2016). OTX015 (MK-8628), a Novel BET Inhibitor, Exhibits Antitumor Activity in Non-Small Cell and Small Cell Lung Cancer Models Harboring Different Oncogenic Mutations. Oncotarget.

[85] Winkle M., El-Daly S.M., Fabbri M., Calin G.A. (2021). Noncoding RNA Therapeutics—Challenges and Potential Solutions. Nat. Rev. Drug Discov..

[86] Rizano A., Margiana R., Supardi S., Narulita P. (2023). Exploring the Future Potential of Mesenchymal Stem/Stromal Cells and Their Derivatives to Support Assisted Reproductive Technology for Female Infertility Applications. Hum. Cell.

[87] Adriansyah R.F., Margiana R., Supardi S., Narulita P. (2023). Current Progress in Stem Cell Therapy for Male Infertility. Stem Cell Rev. Rep..

[88] Fernandes G.F.S., Silva G.D.B., Pavan A.R., Chiba D.E., Chin C.M., Dos Santos J.L. (2017). Epigenetic Regulatory Mechanisms Induced by Resveratrol. Nutrients.

[89] Zhang S., Kiarasi F. (2024). Therapeutic Effects of Resveratrol on Epigenetic Mechanisms in Age-Related Diseases: A Comprehensive Review. Phytother. Res..

[90] Zhang T., Du X., Zhao L., He M., Lin L., Guo C., Zhang X., Han J., Yan H., Huang K. (2019). SIRT1 Facilitates Primordial Follicle Recruitment Independent of Deacetylase Activity through Directly Modulating Akt1 and mTOR Transcription. FASEB J..

[91] Huang Q., Chen S., Chen J., Shi Q., Lin S. (2022). Therapeutic Options for Premature Ovarian Insufficiency: An Updated Review. Reprod. Biol. Endocrinol..

